# Full-Spectrum Sequencing Analysis of RNA TOP-PCR-Amplified Extracellular Vesicle RNAs (EV-RNAs) for Healthy Males

**DOI:** 10.3390/cimb48080838

**Published:** 2026-08-18

**Authors:** Kuo-Ping Chiu, Yu-Shin Nai, Yu-Feng Huang

**Affiliations:** 1 Top Science Biotechnologies Inc., 4F, 50-2 Dingping Rd., Sec. 1, Shiding District, New Taipei City 223, Taiwan; 2 Department of Entomology, National Chung Hsing University, Taichung City 402, Taiwan; ysnai@nchu.edu.tw; 3 Department of Computer Science and Engineering, Yuan Ze University, Taoyuan City 334, Taiwan; yfhuang@saturn.yzu.edu.tw

**Keywords:** RNA-based T oligo-primed polymerase chain reaction (RNA TOP-PCR), extracellular vesicle (EV), extracellular vesicle RNAs (EV-RNAs), exosome, microvesicle

## Abstract

Extracellular vesicle RNAs (EV-RNAs) possess important biological functions and strong therapeutic potential. Their composition, however, is not yet fully understood. To obtain a comprehensive insight of the composition for healthy individuals, we adopted RNA-based T oligo-primed PCR (RNA TOP-PCR) to amplify all EV-RNA species for three healthy males. The constituent sequences were then sequenced and analyzed. The results showed that rRNAs constitute the major species (31.1–43.6%) of EV-RNAs, followed by ncRNAs (including lncRNAs, 4.3–7.9%, and sncRNAs, 0.4–1.2%), Y RNAs (3.9–9.3%), mRNAs (0.7–1.7%), and tRNAs (~0.1%), together with small amounts of mitochondrial tRNA, pseudogenes, and miRNAs (<0.004% over mappable reads). Noticeably, ~40% of the EV-RNAs remain unannotated, while the annotated EV-mRNAs seem to bear a major mission focusing on signal transduction and oxidative phosphorylation. Motif sequences for lncRNA vesicle localization highly agree with previous reports. Among Y RNAs, RNY3 and RNY4 are the major isoforms. Interestingly, many sequences overlap with precursor miRNAs but do not overlap with mature miRNAs. Taken together, RNA TOP-PCR is a robust method suitable for the study of EV-RNAs complexity, and EV-RNAs-mediated intercellular regulation seems to be much more complicated than previously thought.

## 1. Introduction

Extracellular vesicles (EVs), including exosomes and microvesicles, were first known to be subcellular membranous organelles for waste exclusion [1,2]. However, studies over the last two decades have shown that these nanoscale organelles are able to transport molecules, including RNAs, DNAs, proteins, and lipids, to distal cells via blood circulation, so as to exert specific biological functions upon the recipient cells [3,4], of which immunity and signal transduction are among the most intensively studied subjects [5,6,7,8]. Moreover, EVs have been designed to become therapeutic vehicles [9,10,11].

Extracellular vesicle-harbored RNAs (EV-RNAs) play a key role in intercellular communication and regulation, and malfunction may result in diseases, including cancer [12,13,14]. Studies have shown that EV-carried mRNAs can be translated by the recipient cells [15], while miRNAs and mRNAs may cause functional alteration to the recipient cells [15,16]. Y RNAs, a mid-sized non-coding RNA species abundantly present in intercellularly exchangeable EVs, were found to have the ability to maintain RNA stability and play a role in DNA replication and immune regulation [8,17].

Following the 2012 meeting of the International Society for Extracellular Vesicles (ISEV), global efforts have been made to standardize the methods of EV-RNA isolation and preparation [18,19]. Indeed, a number of methods and instruments for EV isolation have been developed [20,21]. However, the quantity of EV-RNAs in body fluids frequently falls below the minimum input requirement of a reliable sequencing analysis or other analytical methods. Additionally, most kits focus on only one type of EV-RNA, further making the understanding of EV-RNAs discrete and not comprehensive, even for healthy individuals [22]. To overcome these shortfalls, an efficient RNA amplification method designed for the systematic analysis of “all” RNA species in the sample is required.

We have expanded TOP-PCR (T oligo-primed PCR) technology [23] to create RNA TOP-PCR (RNA-based TOP-PCR) for a complete analysis of all RNA sequences in the sample. As a proprietary technology (patent ID: PCT/US2020/033929), RNA TOP-PCR converts all single-stranded RNAs into their corresponding cDNAs, which are subsequently amplified by TOP-PCR to produce double-stranded DNA (dsDNA) molecules for a full-spectrum analysis of all RNA sequences in the original sample. The experimental procedure is completed within a single tube to prevent the potential loss of precious nucleic acids caused by isolation and, thus, to include all molecules for a complete analysis. The amplified samples can be subjected to sequencing analysis or other analytical methods.

In more detail, current EV-RNA sequencing requires the following essential steps (listed in sequential order): (1) isolation of EVs or exosomes from liquid biopsies; (2) isolation of RNA from EVs/exosomes, and (3) RNA library preparation followed by sequencing analysis. There are many commercial kits available for each step. For example, certain ultracentrifugation devices and reagent kits are available for vesicle isolation, and many RNA isolation kits have been made many years ago for RNA isolation or for sequencing library preparation. However, RNA TOP-PCR is not designed for EV/exosome or RNA isolation or for sequencing library preparation but for amplification and simultaneous conversion of single-stranded RNAs into dsDNAs for a complete analysis.

Here, we report the characterization of EV-RNAs for three healthy males using RNA TOP-PCR via sequencing analysis. This study aims to conduct a comprehensive analysis of normal EV-RNA composition to provide a fundamental natural EV-RNA background for future research for cohort or diseased samples. Based on the discoveries made in this study, we believe RNA TOP-PCR can be applied to various RNA samples, especially the minute RNAs in, for example, plasma cfRNA, FFPE, or forensic samples.

## 2. Materials and Methods

### 2.1. Ethics Statement

All human materials used in this study were reviewed and approved by Human Subject Research Ethics Committee/Institution Review Board (IRB) at Academia Sinica (AS-IRB02-99065). All three participants (ages between 35 and 45 years old) were in good health and had provided clinical information and consent for the research. All regulatory guidelines were strictly complied throughout the study.

### 2.2. Isolation of Extracellular Vesicles and Purification of EV-RNAs

Whole blood was collected in BD Vacutainer Venous Blood Collection tubes (BD, 367525) from each individual. EVs were isolated with exoRNeasy Serum/Plasma Maxi Kit (Qiagen, Hilden, Germany; cat# 77064), with expected vesicle sizes ranging between 50 and 200 nm in diameter [21]. EV-RNAs samples were quantified with Qubit RNA HS Assay kit (Thermo Fisher Scientific, Eugene, OR, USA; cat# Q32852) and then stored at −70 °C. Fragment Analyzer 5200 System (Agilent Technologies, Santa Clara, CA, USA; cat# M5310AA) using either HS RNA kit (Agilent Technologies, USA; cat# DNF-472) or HS NGS Fragment kit (Agilent Technologies, USA; cat# DNF-474) was also used to estimate the quantity and/or quality of RNA and DNA samples.

### 2.3. RNA TOP-PCR Amplification

RNA TOP-PCR procedure contains two steps: conversion of EV-RNAs to cDNA and DNA amplification by TOP-PCR. It is commercially available. EV-RNAs were subjected to amplification by RNA TOP-PCR (Top Science Biotechnologies, New Taipei City, Taiwan; cat# R02) following the manufacturer’s instructions.

### 2.4. Sequencing Library Preparation and Sequencing

Adapters used in TOP-PCR had to be removed prior to sequencing library construction. To make a sequencing library, ~10 ng of DNA generated from previous steps was treated with 2 units of Thermolabile USER II enzyme (NEB, M5508) in 25 μL of 1xTE buffer (10 mM Tris-HCl pH 8.0, 0.1 mM EDTA) and then incubated at 37 °C for 15 min and held at 25 °C to completely remove the adapters. Illumina sequencing libraries were constructed by using NEBNext Ultra II DNA Library Prep Kit (NEB, Ipswich, MA, USA, E7645) following the manufacturer’s instructions. The sequencing library was quantified with Qubit DNA HS Assay kit (Thermo Scientific, Waltham, MA, USA) and stored at −20 °C.

Sizes were estimated by Agilent Fragment Analyzer, and quantification was measured by Roche LightCycler LC480 II (Roche, Basel, Switzerland) machine using qPCR-based KAPA Library Quantification Kit (Roche, Basel, Switzerland; cat# KK4854). Libraries were sequenced with 2 × 150 bp paired-end (PE) sequencing using HiSeq X Ten (Macrogen, Seoul, Republic of Korea).

### 2.5. Processing of Raw Reads

Potential carryover of adapter sequences formed by P and T-3U oligos were removed from raw reads by Cutadapt software (v1.16) (https://github.com/marcelm/cutadapt, accessed on 20 July 2018). P5 and P7 adapters used for Illumina sequencing were also trimmed by Cutadapt. Software PRINSEQ (v0.20.4) was then used to examine base quality score and the presence of ambiguous base (N) [24]. Read quality was then examined by NGS QC Toolkit with the default quality filtering threshold (QV20 ≥ 70%) [25]. For each step, the minimal read length is 15. FLASH with defined parameters (-m 4 -M 151) was applied to combined paired reads into fragments [26].

### 2.6. Mapping and Sequence Analysis

Quality reads were mapped to human genome GRCh38.p12 using RNA-seq aligner STAR with default parameters [27]. In addition, GENCODE reference annotation (version 29) was employed to identify genes in the human genome [28]. Gene-associated reads were calculated and analyzed for further analysis by feature Counts software (Subread v2.0.0) with default parameters [29]. Post-processing of SAM/BAM files was accomplished with SAMtools [30], and statistics information was generated from BAM files using the Picard tools (https://broadinstitute.github.io/picard, accessed on 2 December 2019). Picard (v2.21.2) is a component integrated within GATK (v4.1.4.1). MEME suite was used for motif search [31] (Command: meme M1.annotated.lincRNA.fasta -dna -oc . -nostatus -time 18000 -mod zoops -nmotifs 10 -minw 6 -maxw 12 -objfun classic -revcomp -markov_order 0).

## 3. Results and Analysis

### 3.1. Workflow

**Figure 1:**
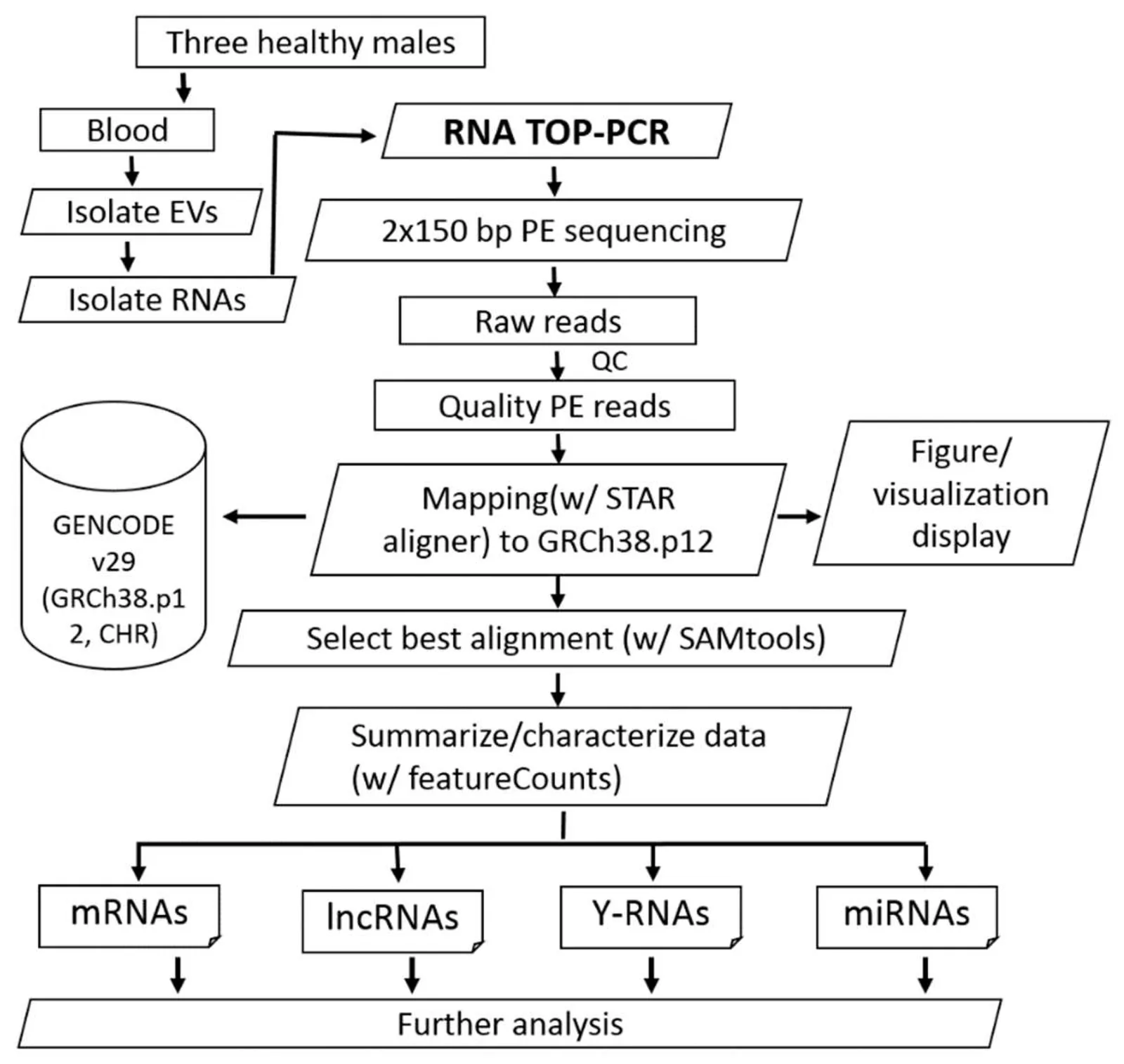
Workflow of EV-RNA sequencing.

A workflow is outlined below to illustrate the process of EV-RNA sequencing (Figure 1). Briefly, EV-RNAs were isolated from extracellular vesicles and subjected to RNA TOP-PCR, which converted RNAs to cDNAs, followed by cDNAs amplification by TOP-PCR. The process was performed in a single tube to prevent the loss of precious material. Adapters in amplified cDNAs were removed by enzymatic digestion, and the cDNAs were sequenced by NGS. Quality reads were mapped to reference genome, and sequence origins were identified based on GENCODE annotation database. Data were then categorized by featureCounts. Sequences of mRNAs, lncRNAs, Y RNAs, and miRNAs were further analyzed.

### 3.2. Library Statistics

Library statistics are shown in Table 1. Only R1–R2-paired mappable reads were used in this study. Comparing to other libraries, the lower ratio of high-quality PE reads observed in M3 was attributed to shorter RNA fragments, a higher percentage of Illumina adapter dimers, and overall poorer sequencing quality, resulting in a greater number of reads being filtered out by the NGS QC Toolkit.

### 3.3. Fragment Size Profiling

**Figure 2:**
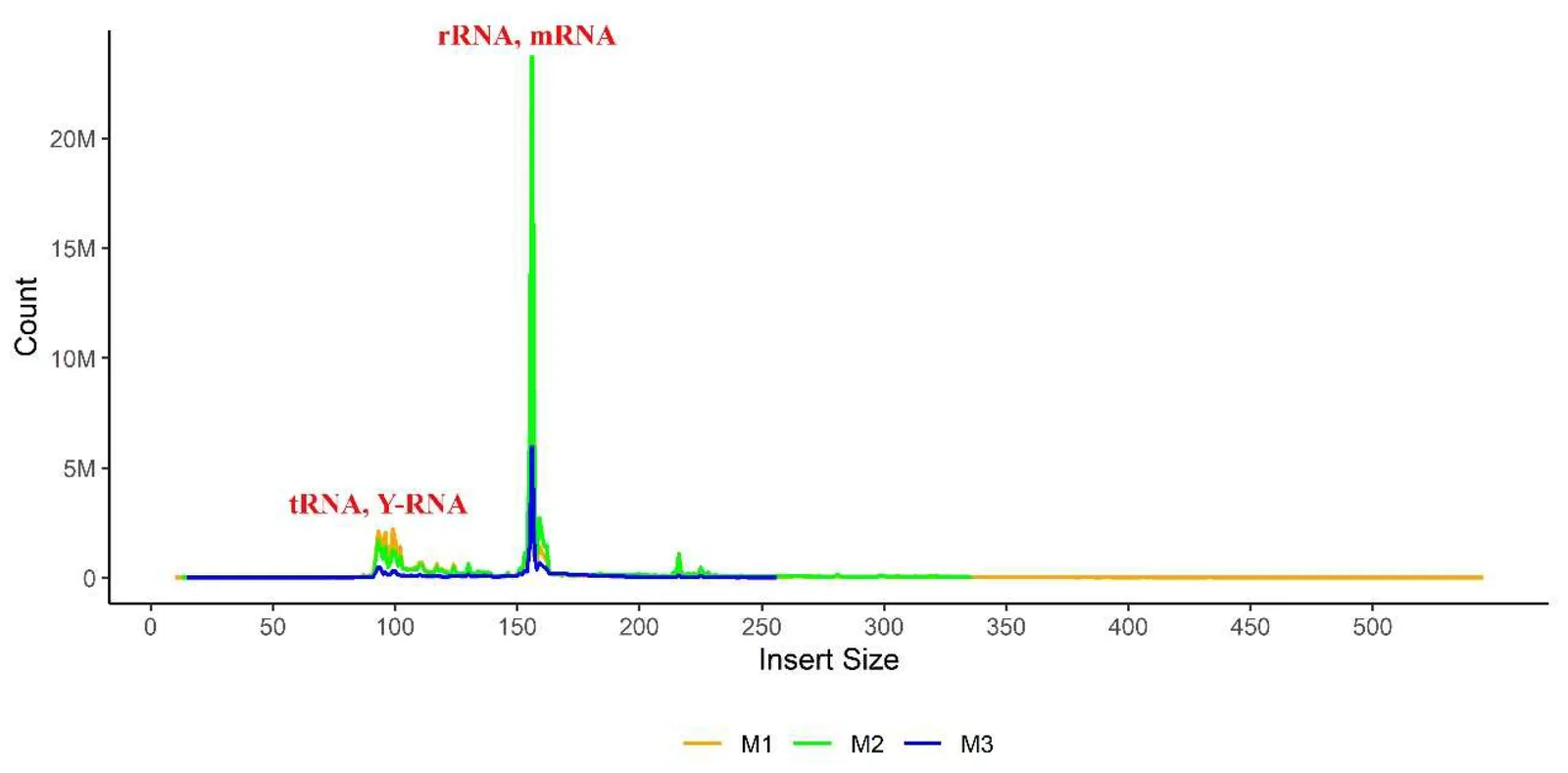
Insert size distribution of all EV-RNAs. (M1, M2, and M3 are the three healthy males that were examined).

Profiling of the fragment sizes in EV-RNA samples revealed two major regions (Figure 2). The major peak ranged between 150 and 170 bases, mainly formed by rRNAs and mRNAs, while the second region ranged between 90 and 110 bases, mainly constituted by Y RNAs and tRNAs (72–80 bases, 87–89 bases (major), and 120–126 bases).

### 3.4. EV-RNA Diversities

As revealed by featureCounts, all EV-RNA collections contain broadly diverse RNA species (Table 2).

### 3.5. Categorization of EV-RNAs

We further associated the EV-RNA species into a few major groups (Table 3). In general, rRNA constitutes the major group, followed by Y RNA. Contrarily, miRNA constitutes the smallest group, likely due to loss in initial RNA isolation or during sequencing library preparation (see the Discussion Section).

### 3.6. Analysis of EV-mRNAs

**Figure 3:**
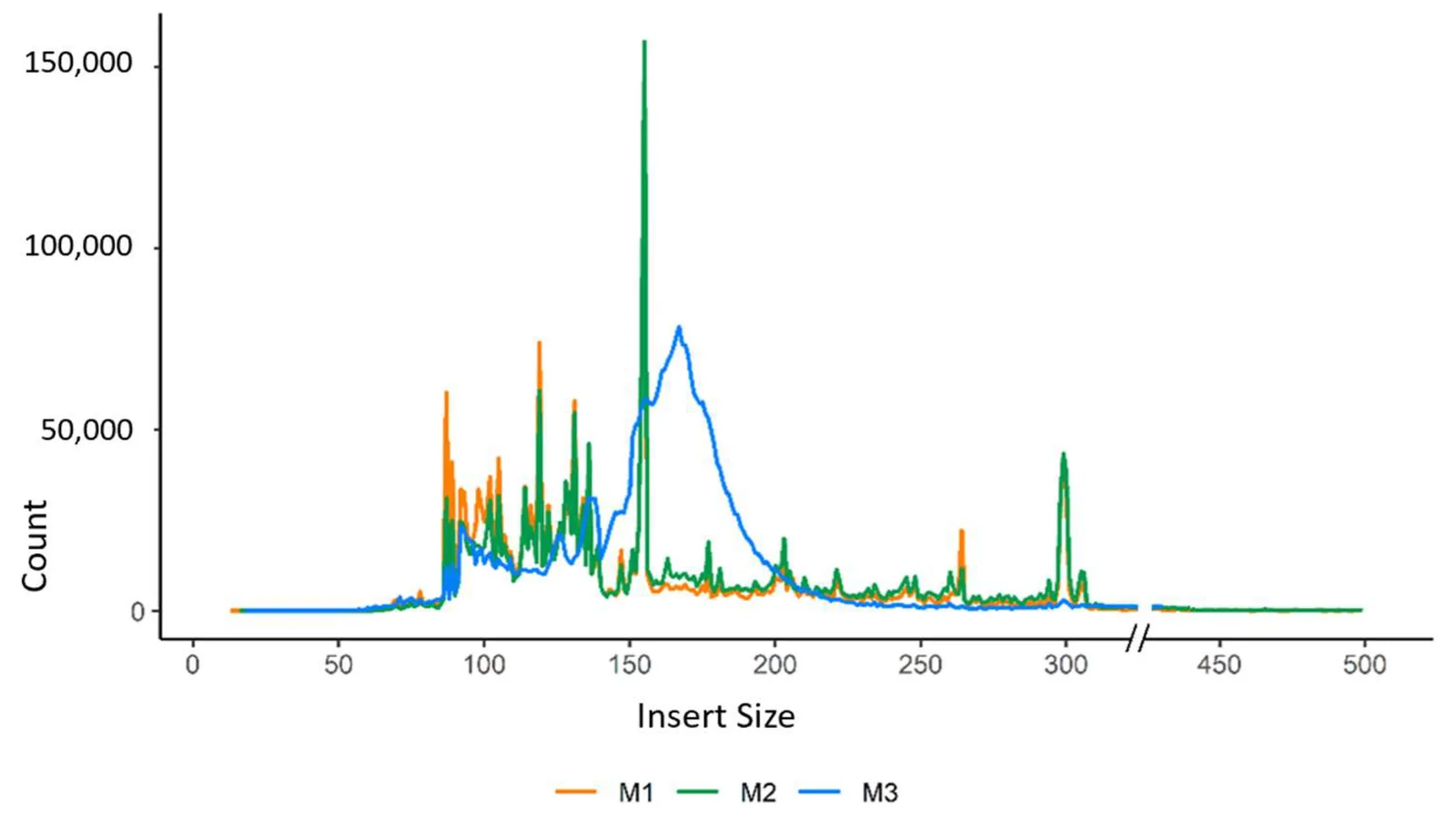
Size distribution of EV-mRNAs. (M1, M2, and M3 are the three healthy males that were examined).

EV-mRNAs, accounting for 0.7–1.7% of mappable sequences, vary broadly in size, with the major population ranging between 100 and 200 bases (Figure 3). Furthermore, the composition may also vary between individuals. Profiles of M1 and M2 are similar. However, the profile of M3 is different from the other two.

**Figure 4:**
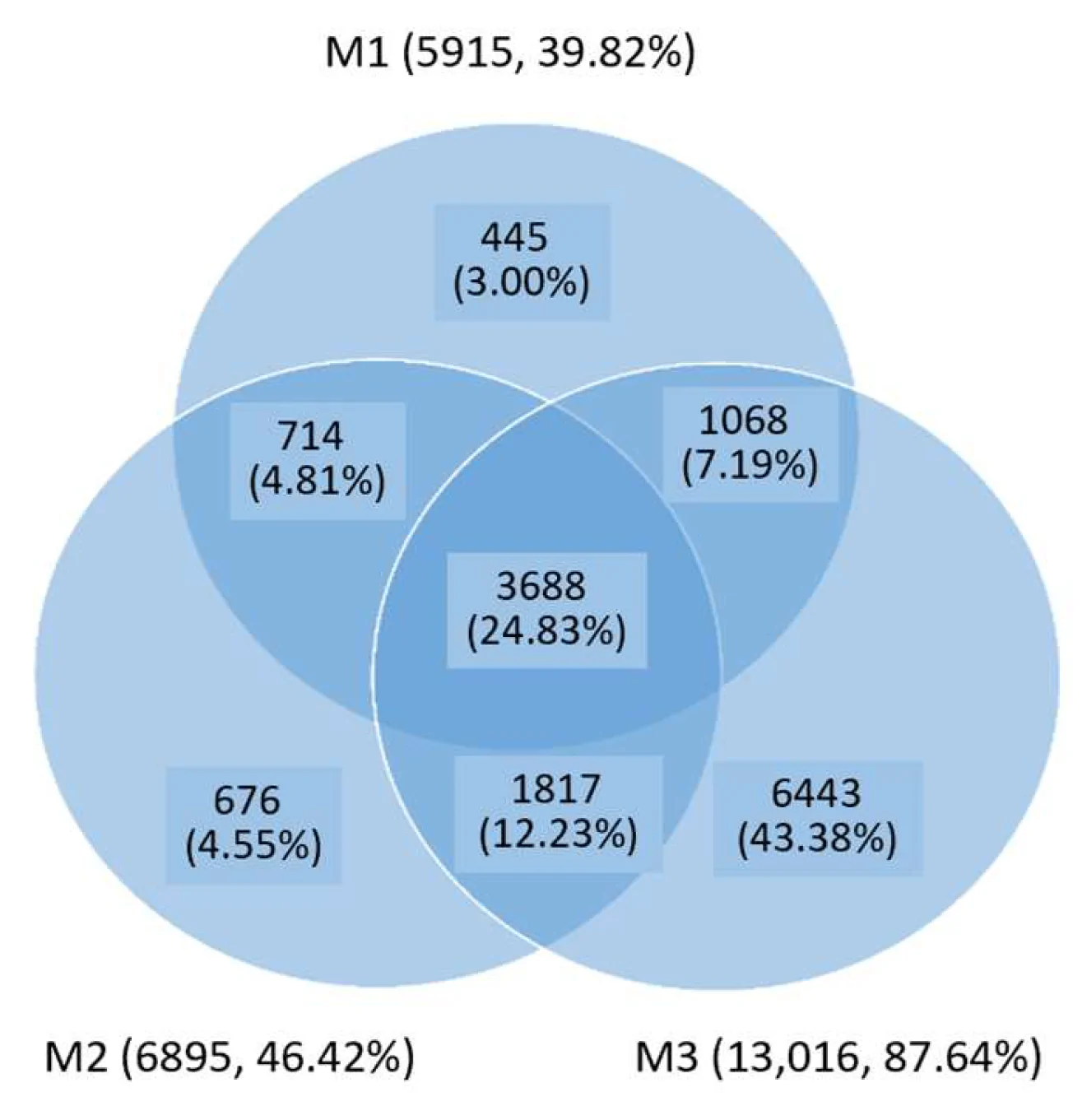
Venn diagram of EV-mRNAs. A total of 14,851 protein-coding genes (mitochondrial, Igb/TCR genes included) were found to be associated with the EV-mRNAs in these three males examined. (% refers to percentage over total number of 14,851 genes. M1, M2, and M3 are the three healthy males that were examined).

The EV-mRNAs in these three healthy males tested were transcribed from a total of ~15,000 protein-coding genes. These individuals shared ~25% overlap between these genes (Figure 4).

### 3.7. Pathway Analysis

Pathway analysis using IPA with common protein-coding genes, i.e., those shared by all three individuals (3688 total), as the input revealed that the top 5 pathways are all associated with signal transduction (Table 4).

Another independent pathway study using IPA together with the top 5000 genes from each individual, ranked by the number of associated PE reads, showed similar results (Table 5).

### 3.8. Top 50 Protein-Coding Genes Associated with EV-mRNAs

To evaluate data reliability through reproducibility, we identified and compared the top 50 protein-coding genes in the three individuals. We found that in any individual, over 50% of the top 50 protein-coding genes are also shared by the other individuals, suggesting a high degree of reproducibility among these individuals (Table 6). High prevalence of mitochondrial-originated sequences also indicated a selectivity of particular mitochondrial sequences, especially those encoding NADH dehydrogenase isoforms.

### 3.9. Unannotated EV-ncRNAs

**Figure 5:**
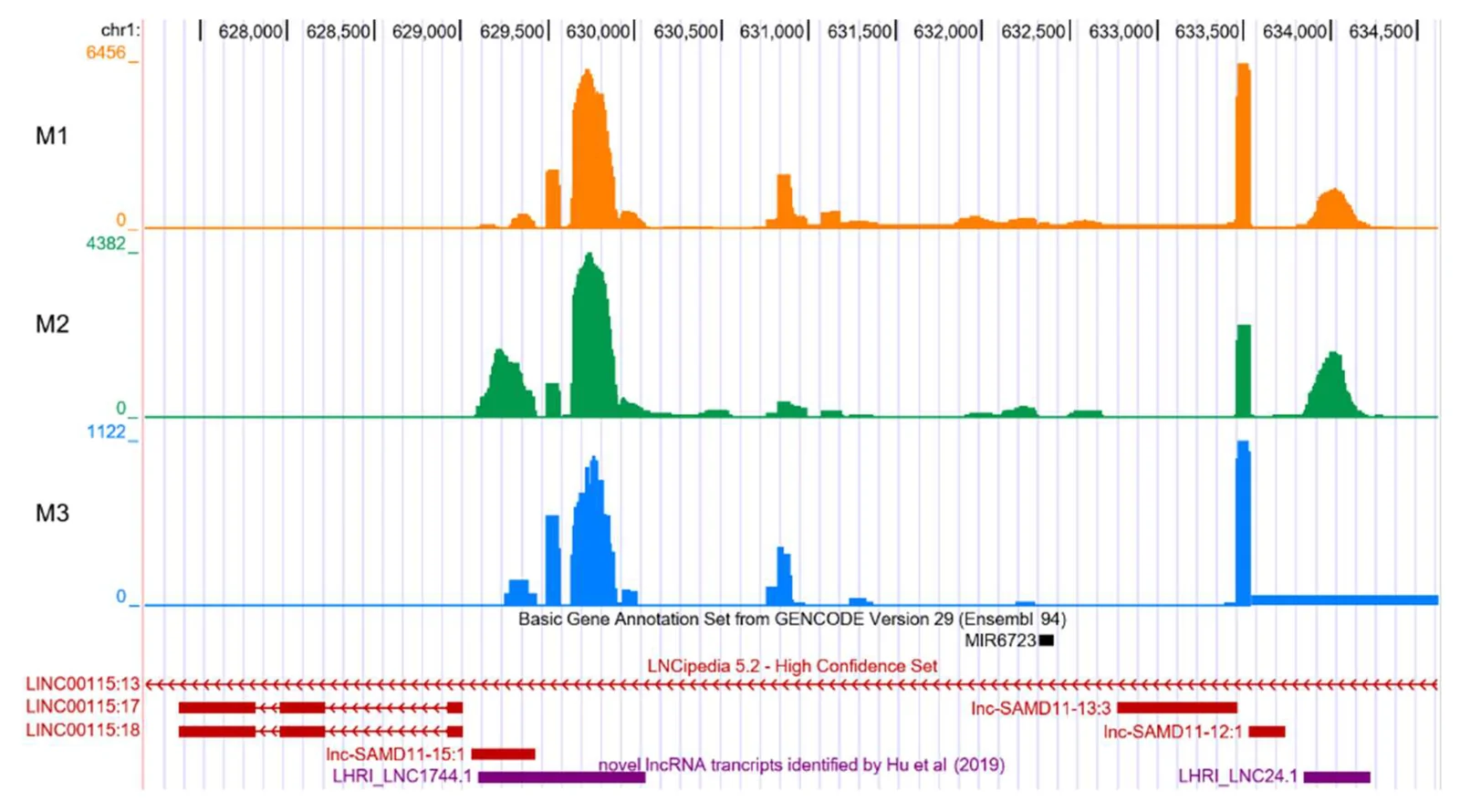
UCSC genome browser display of unannotated ncRNAs identified by RNA TOP-PCR. M1, M2, and M3 showed similar non-coding EV-RNA profiles (three panels on top). MIR6723 (black track, 632,325–632,413), located on the bottom right, is the only transcript annotated by GENCODE in the displayed region. The track below shows high-confidence long non-coding RNAs (red track) from LNCipedia.org (v5.2). The very last track (purple) shows the novel lncRNAs identified and reported by Hu et al. [33]. (M1, M2, and M3 are the three healthy males that were examined).

As shown in Table 3, 39.1–45.6% of EV-RNAs are unannotated. Many of these unannotated ones are in close proximity with the annotated ones (Figure 5). It is not clear whether our reads are actually “mature products” from certain lncRNAs [32], or whether they are fragmented lncRNAs or other RNA species. Furthermore, a considerable fraction of these unannotated EV-ncRNAs is distributed within intergenic regions, suggesting the presence of previously uncharacterized non-coding transcripts.

Our data echoed the report by Hangauer and colleagues who also identified massive novel long intergenic non-coding RNAs (lincRNAs) transcribed from the human genome [34].

It should be noted that lncRNAs are defined as transcripts with lengths over 200 nt and do not encode protein [32]. Various databases collect novel non-coding RNAs, and a significant number of these ncRNAs is unannotated in the GENCODE database. These databases include circBase [35], NONCODE [36], and LNCipedia [37].

### 3.10. Motif Search for EV-Localization of Annotated Sense-Strand EV-lncRNAs

**Figure 6:**
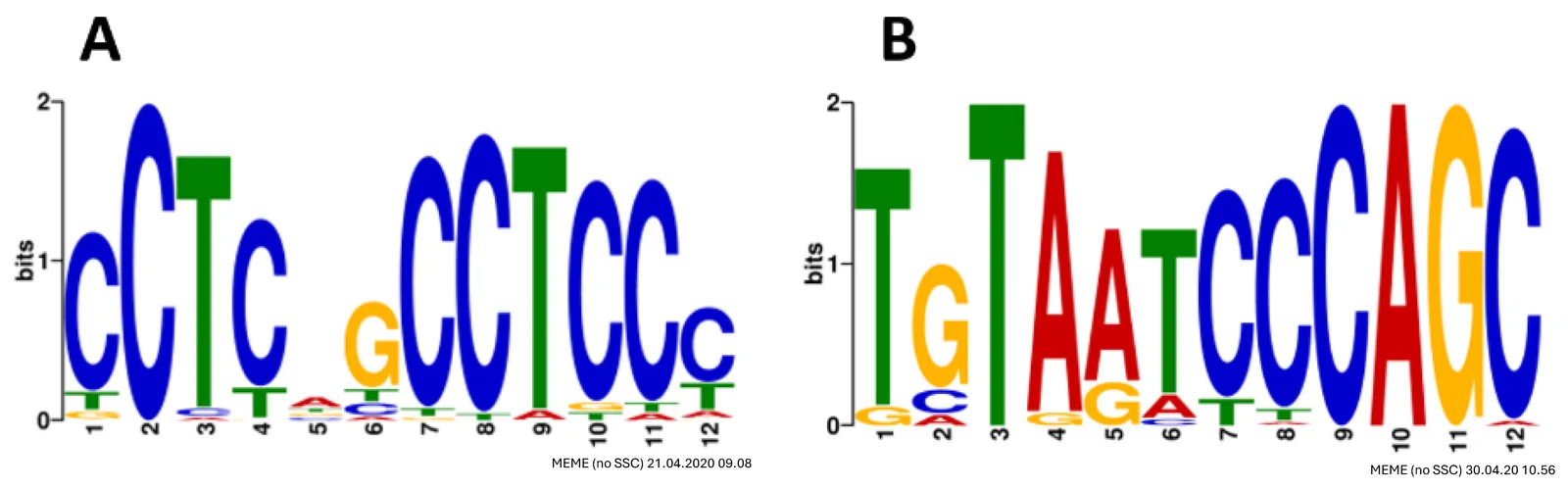
Motifs for vesicle localization of lncRNAs of the annotated sense-strand (**A**) CCUCagGCCUCCC and (**B**) UgUAAUCCCAGC. Four different colors represent four different bases.

A motif search for EV-localization of the annotated sense-strand EV-lncRNAs identified CCUCagGCCUCCC and UgUAAUCCCAGC as the consensus sequences responsible for vesicle localization for EV-lncRNAs (Figure 6A,B).

A similar sequence, CCUCCC, was reported by Ahadi et al. in prostate cancer cell line-derived exosomal lncRNAs [38], and UAAUCCCA was reported by Batagov et al. in glioblastoma cell-derived exosomal RNAs [39]. Additionally, CCUCCC is known to be bound by RNA-binding proteins PCBP1 [40], and UAAUCCCA is known to be bound by YB-1, which is present in EVs [41]. Both RNA-binding proteins have been reported to be EV-associated proteins [42].

### 3.11. Analysis of EV-Y RNA Isoforms

Four Y RNA isoforms have been found in humans, all of which are transcribed by RNA polymerase III. These Y RNAs are known to be repressors of Ro 60-kDa, which is a helical HEAT repeat-containing RNA-binding protein and an initiation factor of DNA replication. Furthermore, biogenesis of small RNAs from Y RNAs is independent of miRNAs [43]. Each type of Y RNA contains a loop domain, upper stem domain, lower stem domain, and polyuridine tail. In the GENCODE annotation database (v29), only RNY1, RNY3, and RNY4 were available; the annotation information of RNY was obtained from NCBI.

**Figure 7:**
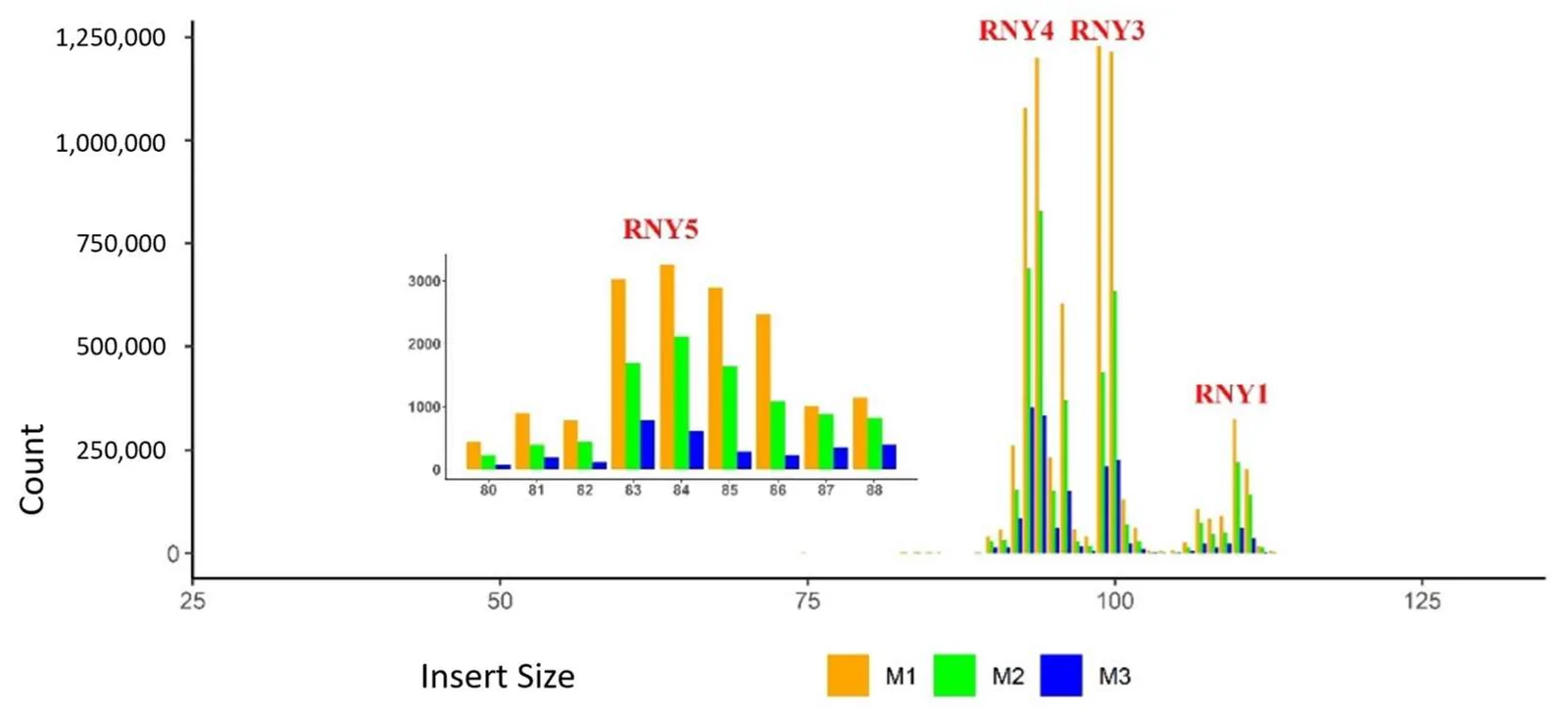
Y RNA profiles among the three healthy males. M1, M2, and M3 are the three healthy males that were examined. RNY5 is enlarged to improved visualization.

Our results show that RNY3 and RNY4 are the major Y RNA species in EVs, which agrees with the previous report by Cambier et al. in 2017 [44], followed by RNY1; moreover, RNY5 is a very minor species, which is in line with the previous report by Driedonks et al. in 2024 [45] (Table 7 and Figure 7). Notably, the relative abundance of EV-Y RNY isoforms may vary between different cell types.

**Figure 8:**
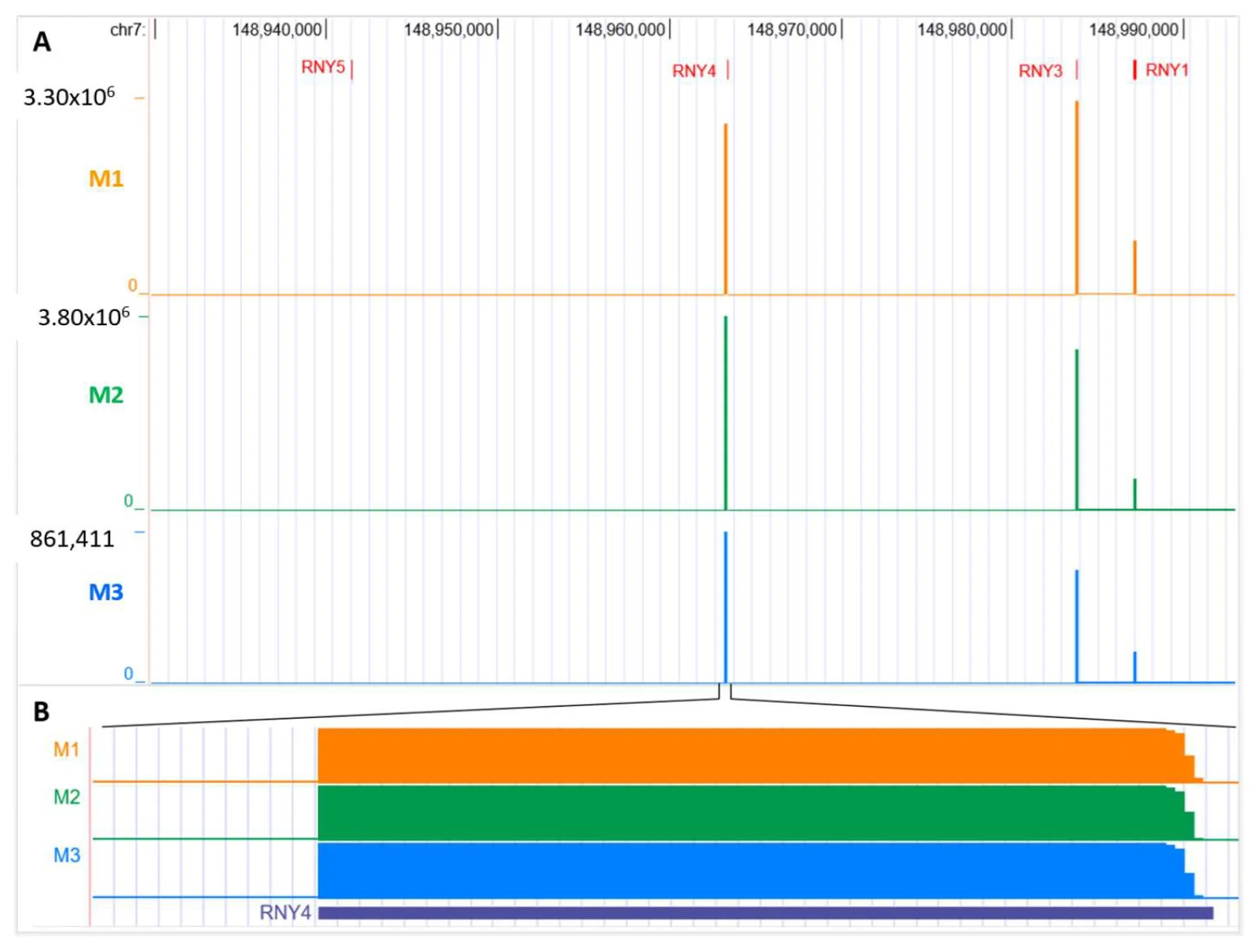
UCSC genome browser display of Y RNA clusters. (**A**) Profiles are exemplified by gene clusters RNY5, RNY4, RNY3, and RNY1 in chromosome 7q36. (**B**) Enlarged displays show the details of sequence lengths of RNY4 among all the three healthy males: M1, M2, and M3. For both panels, 5′ ends are on the left and 3′ ends are on the right.

EV-Y RNA profiles are very similar among all the three individuals examined (Figure 8A). Moreover, at a sequence level, their 5′ ends are extremely uniform, while the 3′ ends may vary, but only regarding a few bases (Figure 8B).

### 3.12. MicroRNA Analysis

**Figure 9:**
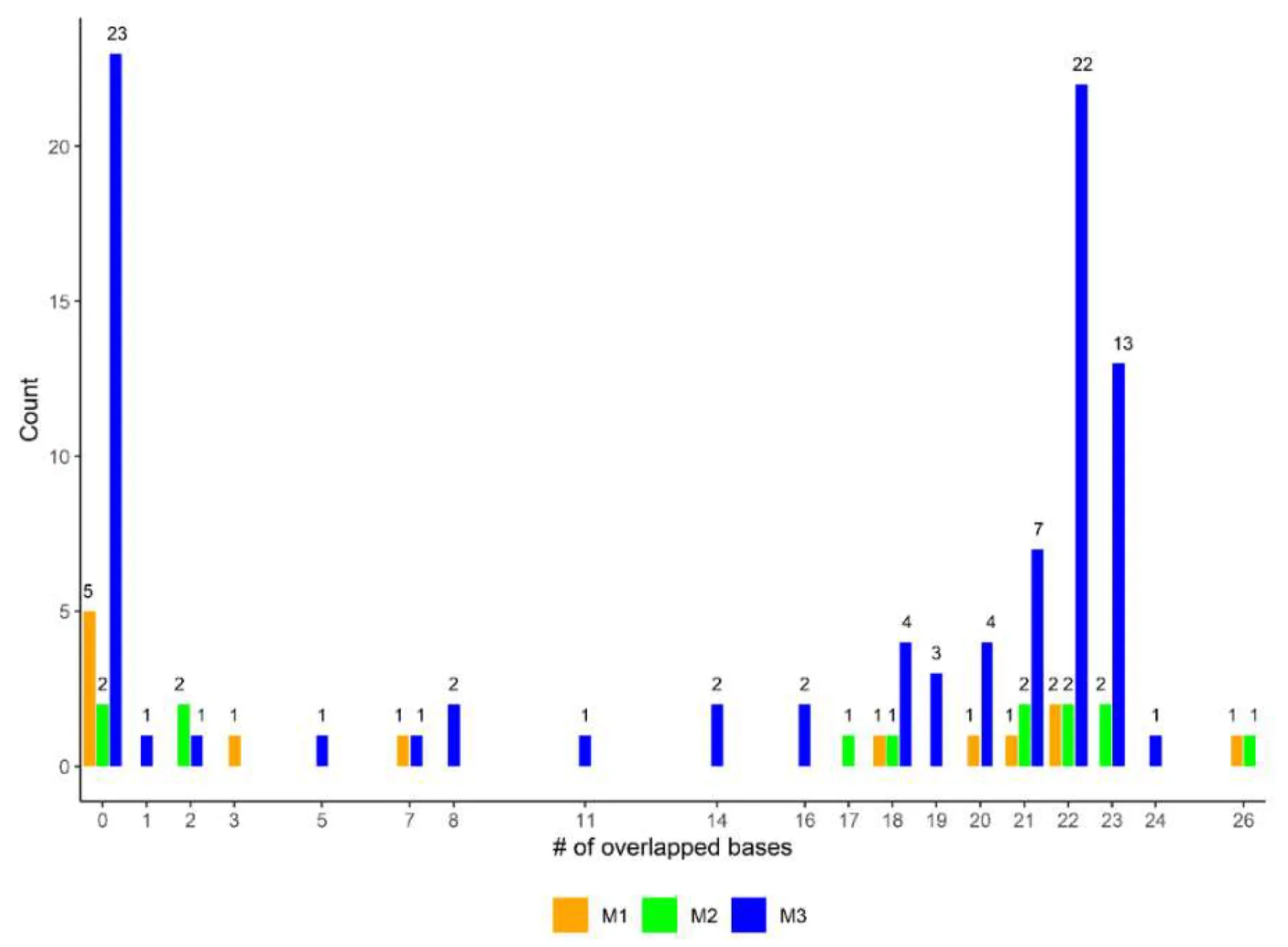
Degree of overlap of EV-miRNAs with mature miRNAs. (M1, M2, and M3 are the three healthy males that were examined).

MicroRNAs represent the smallest population of EV-RNAs. EV-miRNAs were identified by a simple criterion of having at least one base overlapped with pre-miRNAs in the GENCODE database. Information for the annotation of mature miRNAs was obtained from miRBase [46]. Although the criterion may seem loose, the identified EV-miRNAs are likely to cover at least part of the primary or precursor regions that are not present in the mature form. Results showed that most of these EV-miRNAs overlap with their corresponding mature miRNAs (Figure 9).

**Figure 10:**
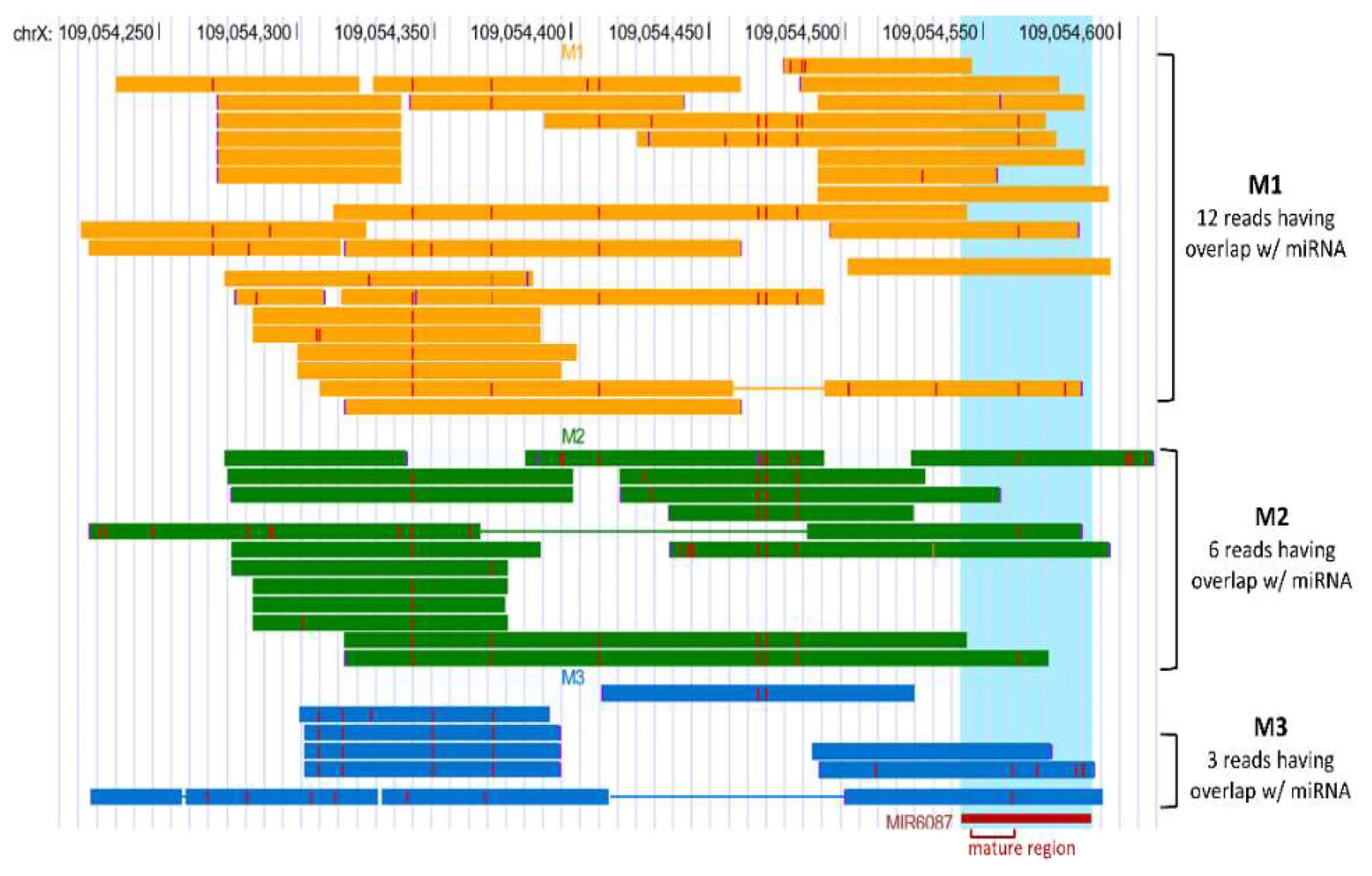
Display of miRNA-associated PE reads, together with known miRNA MIR6087 from the GENCODE (v29) database, using the UCSC genome browser. Reads from individuals M1, M2, and M3 are shown at the top, while MIR6087 (red track) is shown on the bottom right. The mature region of MIR6087 is also labeled. (M1, M2, and M3 are the three healthy males that were examined).

As mentioned above, EV-miRNAs were identified by selecting EV-RNAs with a > 1 base overlap with pre-miRNAs (precursor-miRNAs) in the GENCODE database. Most of such identified EV-miRNAs do have a significant overlap with mature miRNAs (Figure 10).

### 3.13. Comparison of Our Approach with That of Everaert and Colleagues

We compared our results with previous reports (Table 8). Most of the reports, which were also produced from blood plasma-harbored EVs of healthy persons, focused on small or long RNAs in EVs [47,48,49]. Here, we compared our results with the report written by Everaert et al. [50], who, like us, also focused on the analysis of total EV-RNAs.

There were significant differences between our experimental procedure and that employed by Everaert et al. First of all, they pre-excluded rRNAs during the library preparation step; while aiming to compare the EV-RNA profile with them, we masked rRNAs in this study. Secondly, they performed fragmentation on RNAs prior to cDNA synthesis; we, on the other hand, directly used the original EV-RNAs in a single-tube procedure, where no fragmentation or purification was involved until TOP-PCR amplification was completed. Such variations in experimental procedure may be the major reasons causing differences between our outcomes.

## 4. Discussion

Here, we reveal the RNA sequence diversity in normal extracellular vesicles. The sequence diversity is accompanied by a number of interesting phenomena. For example, almost half (~39–46% in the samples tested) of the EV-RNAs are unannotated (Table 3). Furthermore, most unannotated RNAs originated from intergenic regions, making their biological functions even more mysterious and a viable subject of future research. Another interesting example is that an extremely high prevalence of EV-mRNAs is encoded by mitochondria for making NADH dehydrogenase isoforms (Table 6). Since messages carried by EVs are shared by the recipient cells in remote locations, why cells transport these mRNAs through EVs and how cells share these mRNA sequences are peculiarly interesting.

By using annotated lncRNAs in the motif search, we identified an EV-localization consensus signal, which is similar to that reported by Batagove et al. [39], which again demonstrates the selectivity for specific RNA species to be recruited into EVs. Overall, the results suggest that RNA TOP-PCR is a robust method for a full-spectrum analysis of EV-RNA composition.

These interesting phenomena were discovered by proprietary RNA TOP-PCR technology, which converts single-stranded RNAs into their corresponding cDNAs and then uses TOP-PCR to generates double-stranded DNA fragments as the end products. Certain “selective” reagent kits focus on specific RNA species (e.g., miRNAs, mRNAs) and, at the same time, ignore the rest. On the other hand, RNA TOP-PCR was designed for the analysis of “all” RNA species in both regular and minute RNA samples. It possesses a number of advantages inherited from TOP-PCR, including the single-tube procedure, which prevents losing samples by eliminating RNA/cDNA isolation until amplification is completed. Moreover, adapters can be removed after amplification so that the sample can be directly subjected to NGS sequencing analysis or analyzed by conventional methods. The original strands of the RNA sequences can be identified by using an RNA database (e.g., GENCODE or LNCipedia). Moreover, compared to larger RNAs, mature miRNAs are more likely to be underestimated by most protocols, mainly due to the fact that small-sized molecules are more likely to be lost during, and before, sequencing library preparation.

This pilot study recruited only three healthy individuals for analysis. The main purpose of this study was to introduce the application of RNA TOP-PCR technology in EV-RNA analysis while also laying out an experimental procedure for a future cohort study of larger healthy or diseased populations. In fact, we employed a similar strategy in 2018, with which we identified microbial species in early-onset breast cancer (EOBC) patients using plasma cell-free DNA (cfDNA). With only 5 cfDNA samples (2 healthy control and 3 EOBC experimental), we received an enormous response [51].

## 5. Important Implications and Future Directions of This Study

It is important to have an independent approach or method for EV-RNAs analysis. With RNA TOP-PCR, we were able to identify not only the previously reported ncRNAs but also a large amount of novel ncRNA transcription sites in the human genome. Most of the previous studies in this area focused on one or a few species of EV-RNAs; here, we took advantage of the unbiased nature of RNA TOP-PCR and intended to survey all RNA species in EVs. To avoid overestimation of the RNA level, no fragmentation was involved in sample preparation.

As shown in Table 3, a significant portion (~40%) of the EV-RNA remains unannotated. Moreover, many of the EV-miRNA-related sequences, as selected by overlapping with precursor miRNAs (pre-miRNAs), do not even overlap with mature miRNA, making the functions of these miRNA-related sequences extremely mysterious. Future studies are required to unravel the functions of these unannotated and mysterious RNA molecules.

As demonstrated by this study, RNA TOP-PCR provides a simplified procedure for a comprehensive analysis of EV-RNAs. Thus, it has a strong potential to facilitate the study of EV-RNAs and other RNA types, including minute RNA molecules in forensic or fossil samples and cfRNA in patient blood samples. Indeed, RNA TOP-PCR has a great potential to be applied to the study of various RNA samples, including cfRNA, FFPE-derived mRNA, and RNA in forensic samples.

This pilot study demonstrates a robust strategy that allowed us to effectively portray all RNA species in the sample. By applying the same strategy to larger cohorts composed of both healthy and diseased individuals, we should be able to systematically gain comprehensive insights into various important detials regarding not only the composition of EV-RNAs but also the RNAs in various samples. Due to its efficient amplification capability, RNA TOP-PCR is particularly useful for the sequence analysis of RNA samples with minute quantities that are undetectable by Qubit, including the cfRNA, FFPE, and forensic samples mentioned above.

## 6. Conclusions

We identified a large number of EV-mRNAs and found that these mRNA sequences belong to about 15,000 protein-coding genes, which are also involved in signal transduction. There was a high degree of overlap among the top 50 EV-mRNA-coding genes in the three males tested (44% shared by all three plus 8–40% shared by any two of them), suggesting that RNA TOP-PCR and the experimental approach are highly reliable.

Furthermore, most of the top 20 EV-mRNAs encode isoforms of NADH dehydrogenase subunits, which are normally destined to the inner membrane of mitochondria. We suspect that this may be a strategy for an organism to maintain homeostasis during ATP production through the oxidative phosphorylation pathway.

We also identified sequences CCUCagGCCUCCC and UgUAAUCCCAGC as the motifs potentially responsible for EV-lncRNAs vesicle localization. This result is in line with other previous reports [38,39], although in different cell types. Earlier reports also showed that the RNA motif “AGCCC” mediated the strict nuclear localization of lncRNAs [52], and that Alu repeats are able to direct lncRNAs to the nucleus [53].

Results of EV-Y RNAs were in line with the previous reports [54]. Nevertheless, there is a report showing that RNY5 is the most abundant RNY species (human endothelial cell line) as well [55]. It should be noted that Y RNA composition is cell subtype-specific; thus, Y RNA constitution in plasma EVs is influenced by EV-producer cells [8]. Moreover, YB-1 (the RNA-binding protein mentioned above) may play a role in EV-Y RNA vesicle localization [54]. Overall, this study comprehensively demonstrates the sequence diversity of the EV-RNA system, which implies an additional layer of complexity in intercellular gene expression and regulation beyond the intracellular level. Additionally, this study depicts the robustness of RNA TOP-PCR for the comprehensive analysis of EV-RNAs in the cardiovascular system.

Taken together, our results demonstrated that RNA TOP-PCR is a reliable technology for EV-RNA analysis. In conjunction with bioinformatics sequence analysis, it is able to make novel discoveries and new paradigms in biological research. However, future independent validations using biochemical and/or genetic approaches are equally important.

## Figures and Tables

**Table 1:** Library Statistics and Molecular Compositions of EV-RNA Libraries.

Description of Reads		Library IDs of the Individuals Studied		
		M1	M2	M3
Raw PE reads		146,305,550	157,173,135	160,462,457
Quality PE reads	Total	77,564,041	106,202,280	38,429,918
	w/overlap	72,042,366	95,664,333	35,015,882
	w/o overlap	5,521,675	10,537,947	3,414,036
	Mappable	76,264,355	105,021,697	26,958,493

**Table 2:** Summary of Annotated EV-RNAs.

Category	Origins	M1	%	M2	%	M3	%
mRNA	protein_coding	575,271	0.75	730,658	0.70	459,939	1.71
	IG_C_gene	405	0.00	302	0.00	90	0.00
	IG_D_gene	-	-	-	-	-	-
	IG_J_gene	-	-	-	-	-	-
	IG_V_gene	173	0.00	104	0.00	252	0.00
	TR_C_gene	103	0.00	198	0.00	73	0.00
	TR_D_gene	-	-	-	-	-	-
	TR_J_gene	-	-	43	0.00	62	0.00
	TR_V_gene	18	0.00	62	0.00	178	0.00
rRNA	rRNA	23,721,471	31.10	45,737,594	43.55	10,739,589	39.84
tRNA	tRNA	113,933	0.15	69,583	0.07	27,809	0.10
Mt_rRNA	Mt_rRNA	4,684,838	6.14	4,212,871	4.01	437,174	1.62
Mt_tRNA	Mt_tRNA	24,638	0.03	10,163	0.01	2297	0.01
miRNA	miRNA	51	0.00	162	0.00	1030	0.00
Y RNA	Y RNA	7,119,141	9.33	4,068,465	3.87	1,685,919	6.25
Long non-coding RNA	processed_transcript	627	0.00	1256	0.00	5518	0.02
	lincRNA	4,619,995	6.06	8,275,637	7.88	1,104,098	4.10
	3 prime_overlapping_ncRNA	41	0.00	-	-	77	0.00
	antisense	1377	0.00	2520	0.00	25,508	0.10
	non_coding	-	-	-	-	14	0.00
	sense_intronic	146	0.00	355	0.00	2929	0.01
	sense_overlapping	194	0.00	530	0.00	1222	0.00
	TEC	101	0.00	442	0.00	5236	0.02
	known_ncrna	-	-	-	-	-	-
	macro_lncRNA	-	-	1	0.00	301	0.00
	bidirectional_promoter_lncRNA	178	0.00	619	0.00	1027	0.00
	Sum	4,622,659	6.06	8,281,360	7.89	1,145,930	4.25
Small non-coding RNA	snRNA	25,589	0.03	32,939	0.03	8961	0.03
	snoRNA	5422	0.00	6356	0.01	2761	0.01
	misc_RNA	855,309	1.12	721,280	0.69	84,783	0.31
	ribozyme	-	-	-	-	-	-
	sRNA	-	-	-	-	-	-
	scRNA	6	0.00	103	0.00	1	0.00
	scaRNA	905	0.00	1002	0.00	500	0.00
	vaultRNA	-	-	-	-	-	-
Pseudogenes	processed_pseudogene	6736	0.01	6559	0.01	28,022	0.10
	transcribed_processed_pseudogene	290	0.00	717	0.00	3281	0.01
	translated_processed_pseudogene	4	0.00	1	0.00	1	0.00
	unprocessed_pseudogene	13,729	0.02	13,229	0.01	12,435	0.05
	transcribed_unprocessed_pseudogene	1651	0.00	2310	0.00	10,106	0.04
	unitary_pseudogene	-	-	13	0.00	356	0.00
	transcribed_unitary_pseudogene	166	0.00	199	0.00	1669	0.01
	polymorphic_pseudogene	553	0.00	602	0.00	513	0.00
	pseudogene	-	-	-	-	-	-
	rRNA_pseudogene	9286	0.01	12,412	0.01	2399	0.01
	IG_C_pseudogene	-	-	-	-	1	0.00
	IG_J_pseudogene	-	-	-	-	-	-
	IG_pseudogene	-	-	-	-	-	-
	IG_V_pseudogene	7	0.00	-	-	437	0.00
	TR_J_pseudogene	-	-	-	-	-	-
	TR_V_pseudogene	-	-	-	-	24	0.00
	tRNA_pseudogene	-	-	-	-	73	0.00
		41,782,354	54.8	63,909,287	60.9	14,656,665	54.4

%, percentage over number of mappable reads. misc_RNA, RNA lacking sufficient evidence to be classified into established RNA categories.

**Table 3:** Major Groups of EV-RNAs.

	Group	M1		M2		M3	
	Total Mappable Reads	76,264,355	%	105,021,697	%	26,958,493	%
1	rRNA	23,721,471	31.10	45,737,594	43.55	10,739,589	39.84
2	tRNA	113,933	0.15	69,583	0.07	27,809	0.10
3	Mt_rRNA	4,684,838	6.14	4,212,871	4.01	437,174	1.62
4	Mt_tRNA	24,638	0.03	10,163	0.01	2297	0.01
5	mRNA	575,970	0.76	731,367	0.70	460,594	1.71
6	Long non-coding RNA	4,622,659	6.06	8,281,360	7.89	1,145,930	4.25
7	Small non-coding RNA	887,231	1.16	761,680	0.73	97,006	0.36
8	Y RNA	7,119,141	9.33	4,068,465	3.87	1,685,919	6.25
9	miRNA	51	0.00	162	0.00	1030	0.00
10	Pseudogenes	32,422	0.04	36,042	0.03	59,317	0.22
	Annotated total (sum of above)	41,782,354	54.7	63,909,287	60.9?	14,656,665	54.4
	Unannotated total	34,482,001	45.3	41,112,410	39.1	12,301,828	45.6

%, percentage over number of mappable reads; mRNA (protein-coding genes with mitochondrial genes included, Igb, TCR).

**Table 4:** Pathway Analysis Based on 3688 Genes Shared by All Three Individuals.

EV-RNAs		
Pathway	*p*-Value	Overlap
EIF2 Signaling	5.17 × 10^−65^	149/224 (66.5%)
Regulation of eIF4 and p70S6K Signaling	1.82 × 10^−40^	99/157 (63.1%)
mTOR Signaling	7.79 × 10^−36^	112/210 (53.3%)
Integrin Signaling	7.73 × 10^−30^	105/213 (49.3%)
Estrogen Receptor Signaling	2.09 × 10^−28^	136/328 (41.5%)

**Table 5:** IPA Analysis on Each Individual.

Pathway	*p*-Value	Overlap
M1		
EIF2 Signaling	1.05 × 10^−53^	156/224 (69.6%)
Regulation of eIF4 and p70S6K Signaling	7.31 × 10^−36^	107/157 (68.2%)
mTOR Signaling	5.03 × 10^−32^	124/210 (59.0%)
Integrin Signaling	9.38 × 10^−26^	116/213 (54.5%)
ERK/MAPK Signaling	2.26 × 10^−25^	108/193 (56.0%)
M2		
EIF2 Signaling	1.45 × 10^−54^	157/224 (70.1%)
Regulation of eIF4 and p70S6K Signaling	3.51 × 10^−33^	104/157 (66.2%)
Integrin Signaling	5.16 × 10^−27^	118/213 (55.4%)
Molecular Mechanisms of Cancer	1.03 × 10^−25^	177/391 (45.3%)
Estrogen Receptor Signaling	4.57 × 10^−25^	155/328 (47.3%)
M3		
EIF2 Signaling	1.86 × 10^−19^	109/224 (48.7%)
Molecular Mechanisms of Cancer	1.90 × 10^−14^	150/391 (38.4%)
Protein Kinase A Signaling	1.97 × 10^−14^	152/398 (38.2%)
GNRH Signaling	1.15 × 10^−13^	81/173 (46.8%)
Integrin Signaling	9.61 × 10^−13^	92/213 (43.2%)

**Table 6:** Top 50 Protein-Coding Genes Identified in Exosomal RNA Libraries.

	M1		M2		M3	
	Gene	#Reads	Gene	#Reads	Gene	#Reads
1	*MT-ND1*	28,926	*HBB*	21,020	*HBB*	7801
2	*MT-ND4*	24,854	*MT-ND2*	18,885	*HBA2*	3837
3	*MT-ND2*	23,247	*MT-ND1*	17,676	*TBC1D1*	1498
4	*MT-CO1*	17,498	*MT-ND4*	16,545	*MT-ND5*	1423
5	*HBA2*	15,579	*MT-CO1*	15,262	*MT-ND1*	1159
6	*MT-ATP6*	13,163	*HBA2*	12,220	*MT-ND4*	1034
7	*HBB*	12,919	*TMSB4X*	11,023	*MT-ND2*	956
8	*MT-ND5*	12,773	*ACTB*	10,306	*MT-CO1*	950
9	*MT-CO2*	11,395	*MT-ND5*	9440	*TMSB4X*	929
10	*TMSB4X*	10,842	*MT-CO2*	8501	*EEF1A1*	756
11	*PPBP*	10,640	*MT-ATP6*	7932	*ACTB*	714
12	*MT-CYB*	9601	*PPBP*	7673	*B2M*	638
13	*ACTB*	8256	*MT-ND4L*	7514	*SAMHD1*	610
14	*MT-CO3*	7763	*MT-CYB*	6384	*MT-ATP6*	553
15	*MT-ND4L*	7498	*EEF1A1*	6330	*MT-CO3*	527
16	*MTRNR2L12*	6848	*MT-CO3*	5356	*FCGR2A*	523
17	*B2M*	6033	*B2M*	4339	*BNIP3L*	520
18	*MT-ND3*	3229	*MTRNR2L12*	3859	*PPBP*	505
19	*EEF1A1*	2884	*MT-ND3*	2623	*MT-CYB*	499
20	*PF4*	2467	*PF4*	2540	*TENT5C*	463
21	*CAVIN2*	2272	*FTL*	2403	*MT-CO2*	428
22	*TLN1*	2083	*MYH9*	2324	*MT-ND4L*	406
23	*CCL5*	1977	*FLNA*	2265	*TRAK2*	369
24	*MTRNR2L8*	1945	*TLN1*	2015	*CCL5*	369
25	*F13A1*	1933	*NCOA4*	1932	*MTRNR2L12*	365
26	*FLNA*	1923	*PABPC1*	1914	*HBA1*	361
27	*TUBB1*	1721	*ACTG1*	1908	*SYNE2*	357
28	*NAP1L1*	1531	*EEF2*	1771	*SLC25A37*	327
29	*FTL*	1504	*CAVIN2*	1770	*NACA*	319
30	*ACTG1*	1502	*HBA1*	1767	*TPT1*	314
31	*VCL*	1466	*TPT1*	1732	*MT-ND3*	307
32	*YWHAZ*	1441	*RPL13A*	1720	*CACNA1A*	276
33	*MBNL1*	1400	*TXNIP*	1614	*MUC16*	273
34	*MT-ND6*	1379	*NAGK*	1607	*RPL15*	270
35	*NRGN*	1331	*F13A1*	1598	*PF4*	262
36	*HBA1*	1313	*MBNL1*	1545	*ADGRV1*	260
37	*SPARC*	1269	*RPL10*	1438	*TTN*	255
38	*MYH9*	1266	*RPS8*	1415	*NAV1*	252
39	*NCOA4*	1246	*YWHAZ*	1407	*RUBCN*	248
40	*SERF2*	1076	*RPL3*	1348	*RPS8*	247
41	*TAGLN2*	1059	*TUBB1*	1318	*ERGIC1*	245
42	*FTH1*	1023	*NAP1L1*	1306	*FYN*	242
43	*PABPC1*	1006	*MTPN*	1282	*MYT1L*	238
44	*RPS11*	984	*RPS27A*	1224	*COL20A1*	238
45	*MT-ATP8*	976	*VCL*	1133	*PTPRF*	232
46	*RPLP1*	950	*LCP1*	1129	*MACF1*	230
47	*MTPN*	931	*PRKAR2B*	1124	*RPS11*	229
48	*TENT5C*	884	*MT-ATP8*	1107	*PABPC1*	225
49	*HLA-E*	882	*RPS18*	1090	*TAGLN2*	225
50	*BNIP3L*	875	*SLC25A37*	1089	*LDLRAP1*	225
Shared by all 3 males	44%		44%		44%	
Shared by any 2 of the 3 males	40%		16%		8%	

Orange: shared by all three persons; yellow: shared by any two of them. # Number of reads.

**Table 7:** Y RNA Species of All Subjects.

Gene Name	Size	M1	M2	M3
*RNY1*	113	913,042	593,943	179,880
*RNY3*	102	3,301,852	1,573,633	643,852
*RNY4*	96	2,902,204	1,899,113	861,435
*RNY5*	84	2043	1776	752
		7,119,141	4,068,465	1,685,919

Sizes are based on the NCBI RefSeq database.

**Table 8:** Comparison of Plasma-Derived EV-RNA Profiles from Heathy Individuals.

Reference	PURPOSE	Subject	EV/Isolation Method RNA Extraction Method Library Prep. (Read Length) Annotation Database (Alignment Tool)	Results (Only Annotated RNAs Are Included)
**1. Our current study**	**Total EV-RNA profiling**	3	exoRNeasy Serum/Plasma kit (Qiagen) miRNeasy Serum/Plasma Kit (Qiagen) RNA TOP-PCR + NEBNext Ultra II DNA Library Prep Kit (Illumina, San Diego, CA, USA) (2 × 150 bp) GENCODE v29 (STAR)	mRNAs 7.59% lncRNAs 4.3–7.9% small non-coding RNAs 4.96% Y RNAs 43.61% pseudogenes 0.74% miRNAs < 0.004% tRNAs 0.83%
**2. Everaert et al., 2019** [50]	Total RNA profiling	1	Size exclusion chromatography + OptiPrep density gradient centrifugation miRNeasy Serum/Plasma Kit (Qiagen) Stranded SMARTer total RNA-seq kit (Clontech) (2 × 75 bp) GENCODE (STAR)	mRNAs 70% lncRNAs 10.8% miscRNAs 14% pseudogenes 3.3% other 1.6%

## Data Availability

The original sequence data presented in the study are openly available at NCBI with accession number GSE152693 (https://www.ncbi.nlm.nih.gov/geo/query/acc.cgi?acc=GSE152693, accessed on 1 June 2021).
